# Wavelength dependent effects of red and infrared photobiomodulation on neuropathic pain, skeletal muscle integrity, and mitochondrial respiration

**DOI:** 10.1007/s10103-026-05060-7

**Published:** 2026-10-14

**Authors:** Maria Eduarda Rodrigues Santos, Manuel Alejandro Herrera Lopez, Luara Caniato Araujo, Danielle Paula Freitas Bataus Silva, Daniel Ferreira Carreira, Mauricio da Silva Baptista, Marucia Chacur

**Affiliations:** 1 Laboratory of Neuroanatomy Functional of Pain, Department of Anatomy, Institute of Biomedical Science, Universidade de Sao Paulo, Sao Paulo, Brazil; 2 Department of Biochemistry, Institute of Chemistry, Universidade de Sao Paulo, Sao Paulo, Brazil

**Keywords:** Neuropathic Pain, Photobiomodulation, Mitochondria, Chronic Constriction Injury

## Abstract

Neuropathic pain is a chronic and difficult to treat condition affecting up to 10% of the population, significantly impairing quality of life and representing a public health concern. Photobiomodulation (PBMT) has emerged as a promising therapeutic approach, as photons can reach mitochondria and promote beneficial effects, including increased ATP production, reduced oxidative stress and inflammation, and improved mitochondrial metabolism and regeneration. However, gaps remain regarding its effects on mitochondrial dynamics in neuropathic pain. This study investigated the impact of PBMT at two wavelengths (660 nm, red and 904 nm, infrared) on nociceptive behavior (tactile allodynia, mechanical and thermal hyperalgesia), histological alterations, mitochondrial respiration, and protein expression by Western blotting in an experimental neuropathic pain model in male Wistar rats. PBMT reduced mechanical hyperalgesia and tactile allodynia, decreased inflammatory features, and promoted hyporadiance detected by infrared thermography. Chronic constriction of the sciatic nerve significantly reduced mitochondrial respiration in the gastrocnemius muscle. Although PBMT did not restore mitochondrial respiratory capacity, it induced relevant molecular alterations in mitochondrial proteins. Specifically, PBMT increased the expression of OPA1, a key regulator of mitochondrial dynamics, and modulated components of the electron transport chain and ATP synthase in a wavelength-dependent manner. Overall, PBMT modulated nociception and promoted mitochondrial molecular adaptations. These findings suggest that PBMT-induced analgesia may involve mechanisms independent of skeletal muscle mitochondrial bioenergetic restoration. Distinct wavelength-dependent responses further contribute to therapeutic parameter optimization and support future translational and clinical research. Clinical trial number: not applicable.

## Introduction

Pain is defined by the International Association for the Study of Pain (IASP) as “an unpleasant sensory and emotional experience associated with, or resembling that associated with, actual or potential tissue damage” [1]. Chronic pain affects millions of individuals worldwide and represents a substantial personal, social, and economic burden [2], profoundly impairing physical function, emotional well-being, and quality of life [3–5]. Among chronic pain conditions, neuropathic pain, resulting from lesions or diseases of the somatosensory nervous system is particularly disabling and difficult to treat. Injury to peripheral or central nerve fibers disrupts signal transmission and induces maladaptive neuroplasticity, leading to persistent pain, sensory abnormalities, and secondary tissue alterations [4, 5].

Despite its high prevalence, neuropathic pain management remains suboptimal. Pharmacological therapies often provide incomplete relief and may cause adverse effects, tolerance, or limited long-term efficacy [6–8]. These limitations have increased interest in non-pharmacological and adjunctive therapies capable of targeting multiple biological pathways with fewer side effects.

Photobiomodulation therapy (PBMT) has emerged as a promising non-invasive approach for pain modulation and tissue repair. PBMT uses low-intensity light delivered by lasers or light-emitting diodes (LEDs) to influence cellular processes. Light absorption by mitochondrial chromophores, particularly cytochrome c oxidase, enhances mitochondrial respiration, increases ATP synthesis, modulates redox signaling, and influences inflammatory and neuroimmune pathways [9–12]. Through these mechanisms, PBMT exerts analgesic, anti-inflammatory, and regenerative effects in clinical and preclinical [13–15].

Experimental evidence, including studies from our group, demonstrates that PBMT reduces nociceptive behaviors, attenuates inflammation, and promotes neural and musculoskeletal recovery in models of diabetic neuropathy, orofacial pain, osteoarthritis, and peripheral nerve injury [13–21]. Importantly, PBMT parameters, especially wavelength, determine tissue penetration, cellular targets, and biological outcomes. Red light (~ 660 nm) primarily affects superficial tissues, whereas infrared light (~ 900 nm) penetrates more deeply, potentially influencing peripheral nerves and muscles [22–24]. However, systematic comparisons between red and infrared wavelengths in neuropathic pain models integrating behavioral, muscular, and mitochondrial outcomes remain limited.

Neuropathic pain also affects skeletal muscle through denervation, altered neuromuscular signaling, and sustained inflammation, resulting in atrophy, fiber disorganization, and functional impairment [25–28]. The gastrocnemius muscle is particularly vulnerable due to its proximity to the sciatic nerve. Mitochondria are central to muscle homeostasis, regulating ATP production, calcium handling, and redox balance, and mitochondrial dysfunction is implicated in muscle degeneration and persistent pain [29–37].

Although PBMT has been proposed to enhance oxidative phosphorylation, ATP availability, and inflammatory modulation [38–41], direct evidence linking wavelength specific PBMT effects to mitochondrial respiration in chronically injured skeletal muscle remains scarce.

Therefore, this study investigated the effects of PBMT at red (660 nm) and infrared (904 nm) wavelengths in a rat model of chronic sciatic nerve constriction. We evaluated nociceptive behavior, muscle integrity, inflammatory alterations, and mitochondrial respiratory function in the gastrocnemius muscle. We hypothesized that PBMT would attenuate neuropathic pain and muscle degeneration through mitochondrial modulation, with infrared PBMT producing more robust effects due to deeper tissue penetration. By integrating behavioral, structural, and bioenergetic outcomes, this study aims to provide mechanistic insight and support optimization of PBMT protocols for neuropathic pain.

## Materials and methods

### Animals

Male Wistar rats (2 months old; 200–220 g) were used in this study. Behavioral testing and PBMT treatments were conducted in a dedicated behavioral room at the Department of Anatomy animal facility, equipped with soundproofing and controlled ambient temperature (22 ± 2 °C). Animals were housed under a 12-h light/dark cycle with ad libitum access to standard chow and water and were handled in accordance with established guidelines for the use of laboratory animals in pain and nociception research [42]. Animals were randomly assigned to six experimental groups (*n* = 7 per group) as follows: Group 1: SHAM; Group 2: CCI (chronic constriction injury); Group 3: CCI + PBMT 660 nm (constriction group treated with PBMT 660 nm); Group 4: CCI + PBMT 904 nm (constriction group treated with PBMT 904 nm); Group 5: SHAM + PBMT 660 nm (Sham-operated treated with PBMT 660 nm); Group 6: SHAM + PBMT 904 nm (Sham-operated treated with PBMT 904 nm).

### Neuropathic pain model induction

Neuropathic pain was induced using the chronic constriction injury (CCI) model as originally described by Bennett and Xie (1988) [43]. Animals were anesthetized with isoflurane (5% for induction in oxygen at 5 L/min, followed by 2% for maintenance at 4 L/min). After trichotomy of the mid-thigh region, a longitudinal skin incision was made parallel to the femoral axis. The underlying musculature was gently separated to expose the sciatic nerve proximal to its trifurcation. Approximately 10 mm of the sciatic nerve was carefully dissected, and four loose ligatures (4 − 0 chromic gut) were placed around the nerve with ~ 1 mm spacing, ensuring that epineural blood flow was not compromised. The incision was then sutured in layers. Sham-operated animals underwent the same surgical procedure, including nerve exposure, but without ligature placement.

### Photobiomodulation treatment – PBMT

Each photobiomodulation therapy (PBMT) session consisted of irradiation at nine predefined points using a GaAs laser (Laserpulse, IBRAMED, Brazil), applied in direct contact with the skin. The irradiation points were distributed along the surgical region corresponding to the sciatic nerve pathway in the injured limb, ensuring homogeneous light delivery to the target area. Two wavelengths were used: 660 nm (red) and 904 nm (infrared), delivered at a power density of 45 mW. Each irradiation point had a beam spot area of 0.13 cm² and received 0.78 J of energy, resulting in a total energy delivery of 7.02 J per session. The irradiation time per point was 12 s for 660 nm and 18 s for 904 nm, corresponding to a radiant exposure of 6 J/cm². PBMT treatment was initiated on post-injury day 14, during the established neuropathic phase, and administered three times per week on alternate days, for a total of ten sessions.

The SHAM and CCI groups were subjected to identical handling and experimental procedures during behavioral testing and PBMT treatment sessions; however, no laser irradiation was administered, and no energy delivery occurred. All laser parameters are detailed in Table 1.

**Table 1:** Parameters used for photobiomodulation therapy

Brand and type of emitter	Ibramed GaAs
Wavelength (nm)	904 nm and 660 nm
Frequency (Hz)	9500 Hz
Power Density (mW/cm^2^)	45mW
Beam Spot Size at Target (cm^2^)	0.13 cm^2^
Irradiation Time per Point (s)	18s for 904 nm and 12s for 660 nm
Radiant Exposure (J/cm^2^)	6 J/cm^2^
Energy Delivered per Point (J)	0.72 J
Number of Irradiated Points	9 points
Irradiated Area (cm^2^)	1.17 cm2
Application Technique	In contact with the skin
Number and Frequency of Sessions	10 sessions
Total Energy Delivered (J)	7.02 J each session; 70.2 J in total.

### Experimental design

**Fig. 1:**
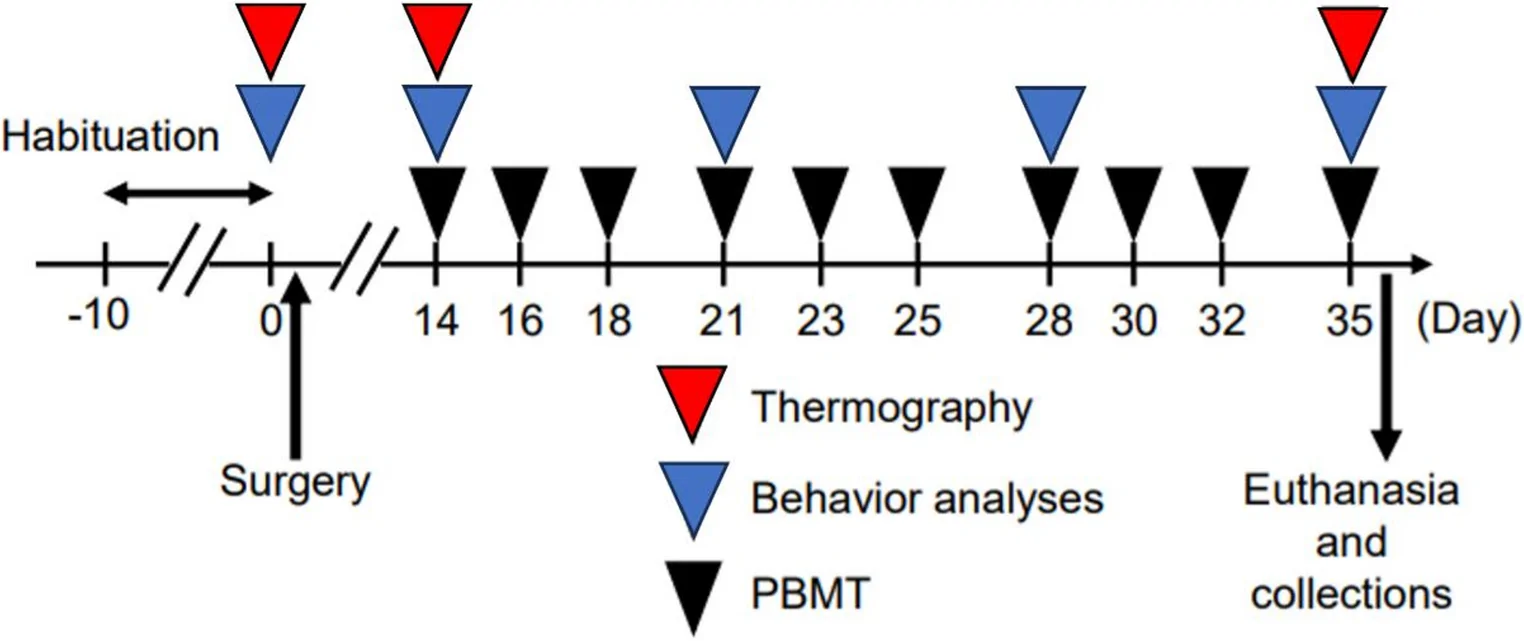
Schematic representation of the experimental timeline, including habituation, baseline assessments, surgical procedures, behavioral testing, photobiomodulation sessions, infrared thermography, and tissue collection

The overall experimental timeline is summarized in Fig. 1. Upon arrival, animals were allowed a 10-day habituation period in the behavioral testing room. On day 0 baseline measurements were obtained prior to any surgical procedure and included assessments of tactile allodynia, mechanical and thermal hyperalgesia, as well as infrared thermography. After day 0, animals underwent either chronic constriction injury (CCI) of the sciatic nerve or the corresponding sham surgery. Behavioral assessments were then conducted weekly on days 14, 21, 28, and 35 post-surgery. Infrared thermography was performed on days 14 and 35 to assess surgery and treatment-related thermal changes. PBMT was initiated on 14D, corresponding to the established neuropathic phase, and administered on alternate days (14, 16, 18, 21, 23, 25, 28, 30, 32, and 35), for a total of 10 sessions. Following the final PBMT session and behavioral evaluations, animals were euthanized for tissue collection and subsequent histological, biochemical, and molecular analyses.

### Behavioral assessments

To evaluate the effects of sciatic nerve constriction and PBMT on pain-related behaviors, all animals underwent baseline assessments prior to any surgical or treatment procedures. After baseline measurements, behavioral testing was performed once week, allowing longitudinal evaluation of nociceptive and thermal responses. The behavioral battery included mechanical hyperalgesia, tactile allodynia, thermal hyperalgesia, and infrared thermography.

#### Mechanical hyperalgesia

Mechanical hyperalgesia was assessed using the paw pressure test (Analgesy-Meter, Ugo Basile^®^, Italy), according to the method of Randall and Selitto (1957) [44]. A progressively increasing force (16 g/s) was applied to the dorsal surface of the ipsilateral hind paw. The nociceptive threshold was defined as the force (grams) at which paw withdrawal occurred.

#### Tactile allodynia

Animals were individually acclimated in acrylic chambers placed on an elevated mesh platform. Tactile allodynia was evaluated using a quantitative Von Frey filament test (Semmes–Weinstein aesthesiometer, Stoelting Co., USA), following the up–down method described by Chaplan et al. (1994) [45]. Filaments (0.407 to 15.136 g) were applied to the plantar surface of the hind paw, and the lowest force eliciting paw withdrawal in two consecutive trials was recorded as the withdrawal threshold.

#### Thermal hyperalgesia

Thermal hyperalgesia was measured using the Hargreaves test [46]. A focused infrared heat stimulus was applied to the plantar surface of the ipsilateral hind paw, and withdrawal latency (seconds) was recorded. A cut-off time of 30.1 s was imposed to prevent tissue damage.

#### Infrared thermography

Infrared thermographic imaging was performed using a FLIR T400sc camera coupled with FLIR ResearchIR 9.0 software. The system provides a resolution of 320 × 240 pixels, a temperature range of − 40 °C to 2,000 °C, thermal sensitivity < 0.05 °C, and accuracy of ± 1% of absolute temperature. Images were acquired at baseline, post-surgery, and after completion of the 10 PBMT sessions, allowing assessment of treatment-related thermal alterations [14].

### Euthanasia and tissue collection

After completion of the photobiomodulation treatment and all behavioral assessments, animals were euthanized by overdose of isoflurane inhalation, administered in a sealed acrylic chamber, followed by decapitation using a guillotine to ensure death, in accordance with institutional and ethical guidelines. Immediately after euthanasia, the gastrocnemius muscle was harvested from the ipsilateral hind limb, extending from the Achilles tendon to the proximal tendon insertion. The gastrocnemius muscle was selected because it is one of the major muscles innervated by the sciatic nerve and is highly susceptible to structural and metabolic alterations following chronic constriction injury. Moreover, its high mitochondrial content and energy demand make it a suitable tissue for evaluating both histological and mitochondrial bioenergetic changes associated with neuropathic pain. The muscle was promptly weighed using an analytical balance to determine muscle mass. Tissue samples were then processed for mitochondrial respiration analysis and histological evaluation using hematoxylin–eosin staining.

### Oxygen consumption analysis

Following three weeks of PBMT, mitochondria were isolated from the gastrocnemius muscle according to the protocol described by Gostimskaya and Galkin (2010) [47], with modifications previously established in our laboratory [48]. All procedures were performed at 4 °C to preserve mitochondrial integrity. Briefly, muscle samples were washed in PBS supplemented with 10 mM EDTA, minced into small fragments, and incubated for 10 min in washing buffer (0.3 M sucrose, 10 mM HEPES, 2 mM EGTA, 1 mM EDTA; pH 7.2) containing 0.125 mg/mL trypsin. Tissues were then homogenized and incubated for an additional 5 min in the same buffer. The enzymatic reaction was terminated by dilution with 0.2% bovine serum albumin (BSA). The homogenate was centrifuged at 600 × g for 15 min, and the resulting supernatant was centrifuged at 8,500 × g to obtain the mitochondrial pellet. The pellet was resuspended, and protein concentration was determined using the BCA method. Oxygen consumption rates were normalized to the total protein content added to the respirometry chamber. Mitochondrial respiration was measured using a high-resolution Clark-type respirometer (Oroboros Oxygraph-2k) in a 1 mL chamber, under constant stirring at 37 °C. Respiration was assessed in a standard buffer containing 250 mM sucrose, 100 µM EGTA, 1 mg/mL BSA, and 10 mM HEPES (pH 7.2, KOH). Following established protocols, mitochondria (75 µg/mL) were energized with succinate (2 mM) in the presence of rotenone (1 µM) to prevent reverse electron transport [48, 49]. Distinct mitochondrial respiratory states were evaluated by sequential addition of specific modulators. Basal respiration (state 2) was recorded initially with substrates (Succinate plus rotenone), followed by addition of ADP (1 mM) to assess state 3 (ADP-stimulated respiration). Oligomycin (1.25 µg/mL) was then added to inhibit ATP synthase and determine state 4 respiration. Finally, maximal uncoupled respiration (state 3U) was observed by addition of CCCP (1 µM). The respiratory control ratio (RCR) was calculated as the ratio of state 3 to state 4 respiration. Data acquisition and calculations were performed using Oroboros DatLab software (version 7.4), and statistical analyses were conducted using Origin DatLab 2022b.

### Staining technique: Hematoxylin-eosin and fractal dimension

After collecting and weighing of the gastrocnemius muscle, the samples were coated with neural talc (Tragacanth - Sigma-Aldrich^®^), smeared with biological glue (Tissue Tek - Sakura^®^), and temporarily stored in a freezer (-80 °C) for histological sectioning using a cryostat (Leica CM3050). Cross sections of the muscle belly 10 μm thick were made and stained using the Hematoxylin-Eosin (HE) technique to mark the cellular components. The images were obtained using a light microscope (Nikon, model E800). Fractal dimension analysis is an approach by quantifying the distribution of pixels in the image. The gastrocnemius muscle was analyzed, and the images were obtained by light microscopy and processed using ImageJ^®^ software. Values closer to 2 indicate greater complexity in the organization of the components, expressed on a standard scale from 0 to 2.

### Western blotting assay

Protein expression in isolated mitochondrial fractions was analyzed by Western blotting. Protein concentration was determined using the Bradford assay. Equal amounts of protein were separated by SDS–PAGE on 10% polyacrylamide gels and transferred onto nitrocellulose membranes (0.2 μm pore size; Millipore). Membranes were briefly stained with Ponceau S to verify equal protein loading and transfer efficiency. Ponceau S staining was also used as the loading control for normalization during densitometric analysis. After washing, membranes were blocked for 1 h at room temperature with 5% bovine serum albumin (BSA) in Tris-buffered saline containing 0.1% Tween-20 (TTBS). Membranes were then incubated overnight at 4 °C with the following primary antibodies: OPA1 (1:500, rabbit polyclonal; Abcam, ab42364), and Total OXPHOS (1:1000; Rodent WB Antibody Cocktail, Thermo Fisher Scientific/Invitrogen). After washing with TTBS, membranes were incubated for 1.5 h at room temperature with horseradish peroxidase–conjugated secondary antibodies: donkey anti-rabbit IgG or donkey anti-mouse IgG (both 1:15,000; Jackson ImmunoResearch). Protein bands were visualized using an enhanced chemiluminescence (ECL) detection kit (Amersham Biosciences, USA) and captured with a UVITEC imaging system (United Kingdom). Densitometric analysis of immunoreactive bands was performed using ImageJ software (NIH, USA). Band intensities were normalized to the corresponding Ponceau S staining intensity, and protein expression levels were expressed as normalized target protein/Ponceau S intensity values.

### Statistical analysis

Data are presented as mean ± SEM or SD, as indicated. Statistical analyses for behavioral assays, thermal analysis, hematoxylin–eosin staining, fractal dimension analysis, muscle weight, and Western blot data were performed using GraphPad Prism version 8.0 (GraphPad Software, San Diego, CA, USA). Depending on the experimental design, data were analyzed using one-way or two-way analysis of variance (ANOVA), followed by Tukey’s multiple comparisons test when appropriate. Data from high-resolution respirometry experiments were analyzed using Origin DatLab 2022b. Statistical significance was considered at *P* < 0.05.

## Results

### Photobiomodulation differentially modulates nociceptive behaviors after CCI

**Fig. 2:**
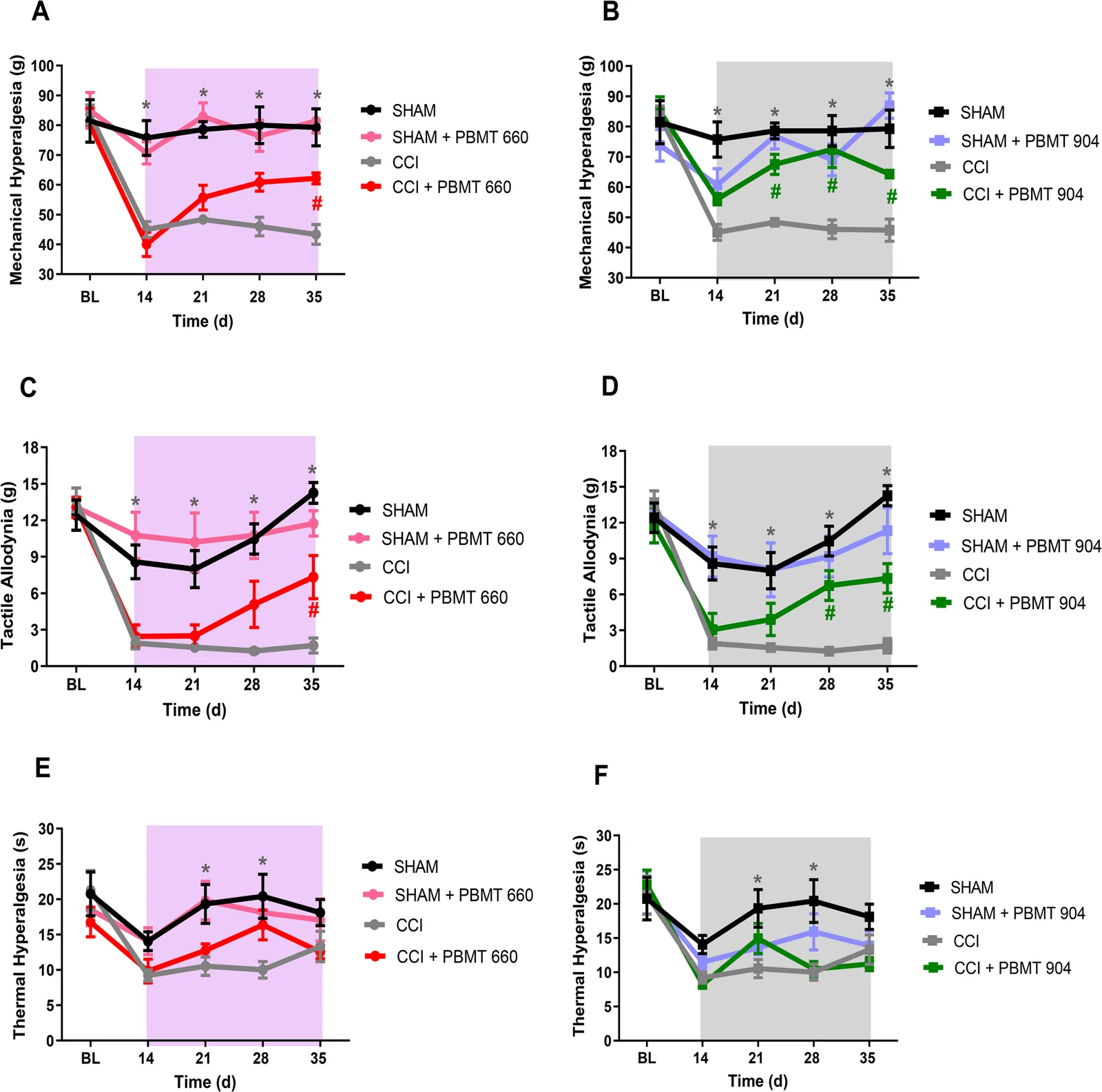
Effects of red and infrared PBMT on nociceptive behavior in CCI animals. Mechanical hyperalgesia (**A**–**B**), tactile allodynia (**C**–**D**), and thermal hyperalgesia (**E**–**F**) were evaluated following sciatic nerve chronic constriction injury (CCI). PBMT was applied at 660 nm (A, C, E) or 904 nm (B, D, F). Measurements were obtained at baseline (BL) and on days 14, 21, 28, and 35 after injury. Data are expressed as mean ± S.E.M. Statistical analysis was performed using two-way ANOVA followed by post hoc tests. *P < 0.05 SHAM vs. CCI ; #*P* < 0.05 CCI + PBMT 660 and CCI + PBMT 904 vs. CCI. *n* = 7 animals per group

To evaluate the effects of PBMT on nociceptive behavior following CCI injury, animals were subjected to behavioral tests assessing mechanical hyperalgesia (Fig. 2A–B), tactile allodynia (Fig. 2C–D), and thermal hyperalgesia (Fig. 2E–F). Following CCI, animals exhibited a marked reduction in pain thresholds compared with baseline values and sham-operated controls, confirming the establishment of a robust neuropathic pain state (Fig. 2).

Treatment with PBMT at 660 nm resulted in a significant increase in pain threshold only at the end of the treatment protocol, on day 35 (F (12, 117) = 3,415, *P* < 0.001, Fig. 2A). In contrast, PBMT at 904 nm produced earlier and sustained antinociceptive effects (F (12, 121) = 4,047, *P* < 0.0001), with significant improvements observed from day 21 through day 35 (Fig. 2B; D21 #*P* < 0.01; D28 #*P* < 0.001; D35 #*P* < 0.05). No significant differences were detected between SHAM and SHAM + PBMT groups.

A similar temporal profile was observed for tactile allodynia. PBMT at 660 nm induced a significant improvement only on day 35 (F (12, 87) = 4,525, *P* < 0.0001, Fig. 2C), whereas PBMT at 904 nm promoted an earlier reversal, with significant recovery detected from day 28 and maintained through day 35 (F (12, 93) = 3,303, *P* < 0.001, Fig. 2D).

In contrast, thermal hyperalgesia was not significantly modified by PBMT. CCI animals exhibited reduced withdrawal latencies compared to sham controls and baseline values at days 21 and 28 (Figs. 2E–F; ***P < 0.001), and neither 660–904 nm PBMT significantly reversed thermal hypersensitivity when compared to untreated CCI animals.

### Infrared thermography reveals PBMT-induced modulation of local thermal changes following sciatic nerve constriction

**Fig. 3:**
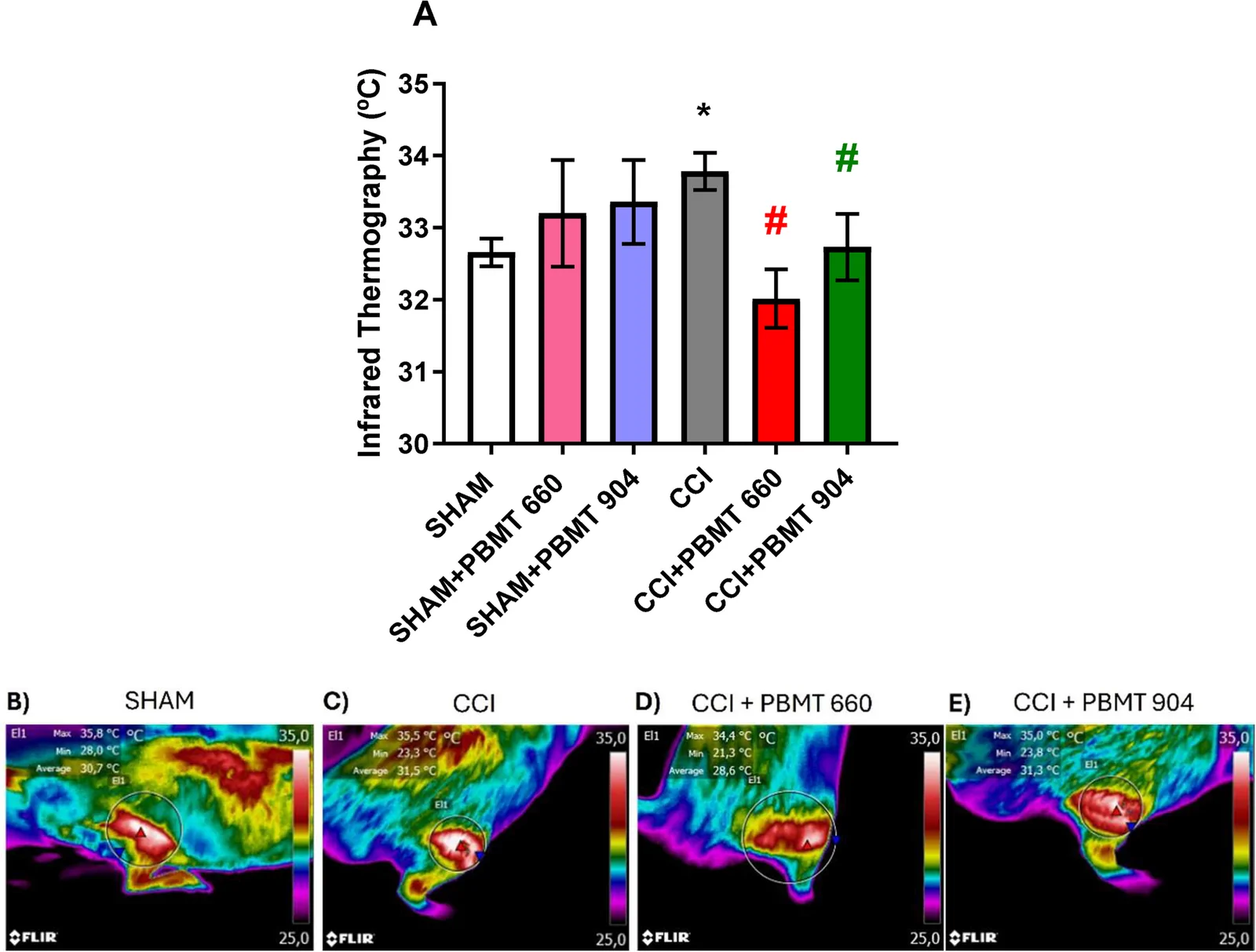
PBMT attenuates injury-induced thermal changes following sciatic nerve constriction. **A**) Quantitative analysis of surface temperature variation (°C) between groups. CCI animals exhibited a significant increase in local temperature compared to SHAM controls (*P < 0.001). PBMT treatment significantly reduced this temperature increase at both wavelengths (#*P* < 0.001 for CCI + PBMT 660 nm vs. CCI; #*P* < 0.001 for CCI + PBMT 904 nm vs. CCI). **B **- **E**) Representative infrared thermographic images of the ipsilateral hind paw (injured side) from SHAM, CCI, CCI + PBMT 660 nm, and CCI + PBMT 904 nm groups after the treatment period. Thermographic images were analyzed using FLIR ResearchIR 9.0 software. Data are expressed as mean ± S.E.M.; *n* = 7 animals per group

Animals subjected to chronic constriction injury (CCI) exhibited a significant increase in surface temperature of the ipsilateral hind limb when compared to sham-operated controls, indicating injury-associated thermal alterations (F (3, 19) = 6,945, *P* < 0.001, Fig. 3A). Treatment with PBMT significantly attenuated this temperature increase at both wavelengths tested. Animals treated with PBMT at 660 nm and 904 nm showed lower surface temperatures compared to untreated CCI animals (Fig. 3A; *P* < 0.001 for CCI vs. CCI + PBMT 660; *P* < 0.001 for CCI vs. CCI + PBMT 904). The laser treated control groups (SHAM + PBMT 660 and SHAM + PBMT 904) showed an increase in hyperradiance compared to the untreated control group (SHAM), as observed in the graph, however this difference was not statistically significant. Likewise, no statistical differences were observed between these groups and the CCI group (Fig. 3A; *P* < 0.57 for SHAM vs. SHAM + PBMT 660; *P* < 0.85 for SHAM vs. SHAM + PBMT 904; *P* < 0.75 for CCI vs. SHAM + PBMT 660; *P* < 0.90 for CCI vs. SHAM + PBMT 904).

Representative infrared thermographic images are shown in Fig. 3B-E, illustrating thermal patterns in animals from the SHAM, CCI, CCI + PBMT 660, and CCI + PBMT 904 groups. These images qualitatively demonstrate increased thermal signals in the ipsilateral paw following CCI and their attenuation after PBMT treatment, corroborating the quantitative analysis.

### Macroscopical changes of muscle integrity following PBMT

**Fig. 4:**
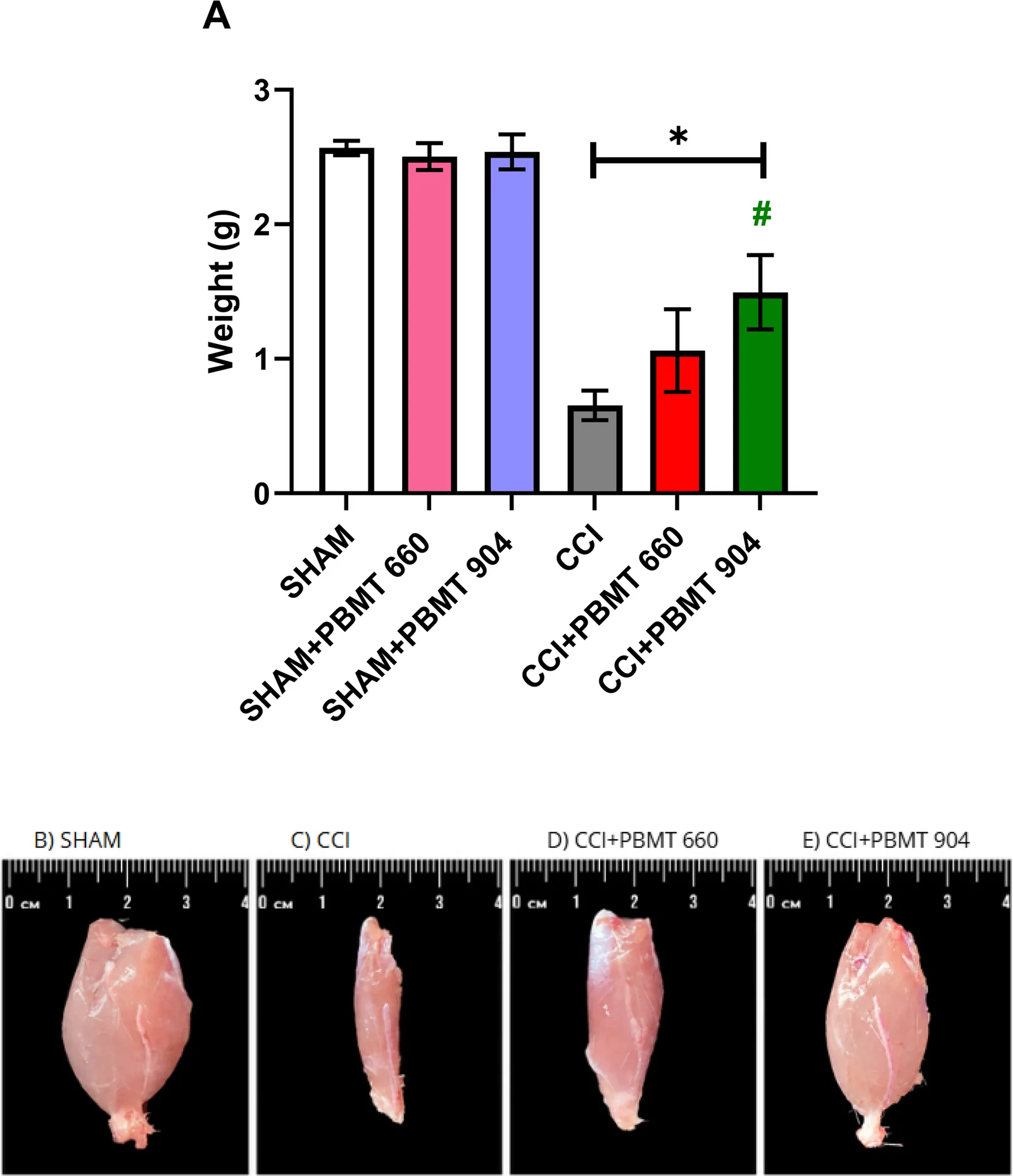
Infrared PBMT (904 nm) attenuates gastrocnemius muscle atrophy induced by sciatic nerve constriction. **A**) Quantitative analysis of muscle weight demonstrates a significant reduction in the CCI group compared to SHAM, SHAM + PBMT 660, and SHAM + PBMT 904 groups (*P < 0.0001). No significant difference was observed between CCI and CCI + PBMT 660. In contrast, treatment with infrared PBMT (904 nm) significantly attenuated muscle atrophy, with the CCI + PBMT 904 group exhibiting greater muscle mass than CCI (#*P* < 0.05). **B **– **E**: Representative images of gastrocnemius muscles illustrate marked atrophy in CCI animals compared to SHAM controls. Data are expressed as mean ± S.E.M. and were analyzed by one-way ANOVA followed by post hoc testing. *n* = 5–7 animals per group

Given the behavioral improvement and attenuation of nociceptive responses observed in PBMT-treated CCI animals, together with the absence of local thermal increases, we next evaluated gastrocnemius muscle integrity as an indicator of peripheral tissue preservation. Muscles were collected and weighed for quantitative analysis. Quantitative analysis revealed a significant reduction in muscle mass in CCI and CCI + PBMT animals compared to SHAM and SHAM-treated groups (F (5, 33) = 18,99, *P < 0.001). Treatment with PBMT at 660 nm did not significantly restore muscle weight relative to untreated CCI animals. In contrast, infrared PBMT (904 nm) significantly attenuated muscle atrophy, with the CCI + PBMT 904 group displaying greater muscle mass than the CCI group (Fig. 4A; #*P* < 0.05). Representative images further illustrate these findings, showing marked gastrocnemius muscle atrophy in CCI animals compared to SHAM controls, while partial preservation of muscle integrity is evident in animals treated with PBMT at 904 nm (Fig. 4B-E).

The percentage analysis of muscle mass reinforces these findings. It was observed that the CCI group showed an approximate 75% reduction in muscle mass compared to the controls, evidencing significant atrophy resulting from nerve injury. The CCI group treated with PBMT 660 nm showed a 59% reduction. In contrast, the CCI group treated with PBMT 904 nm showed a 42% reduction, demonstrating considerable attenuation of atrophy when compared to the untreated CCI group. This percentage difference suggests that the infrared laser exerted a more pronounced protective effect on the integrity of the gastrocnemius muscle, contributing to tissue preservation even in the presence of neuropathic injury.

#### Hematoxylin-eosin and nuclear fractal dimension

**Fig. 5:**
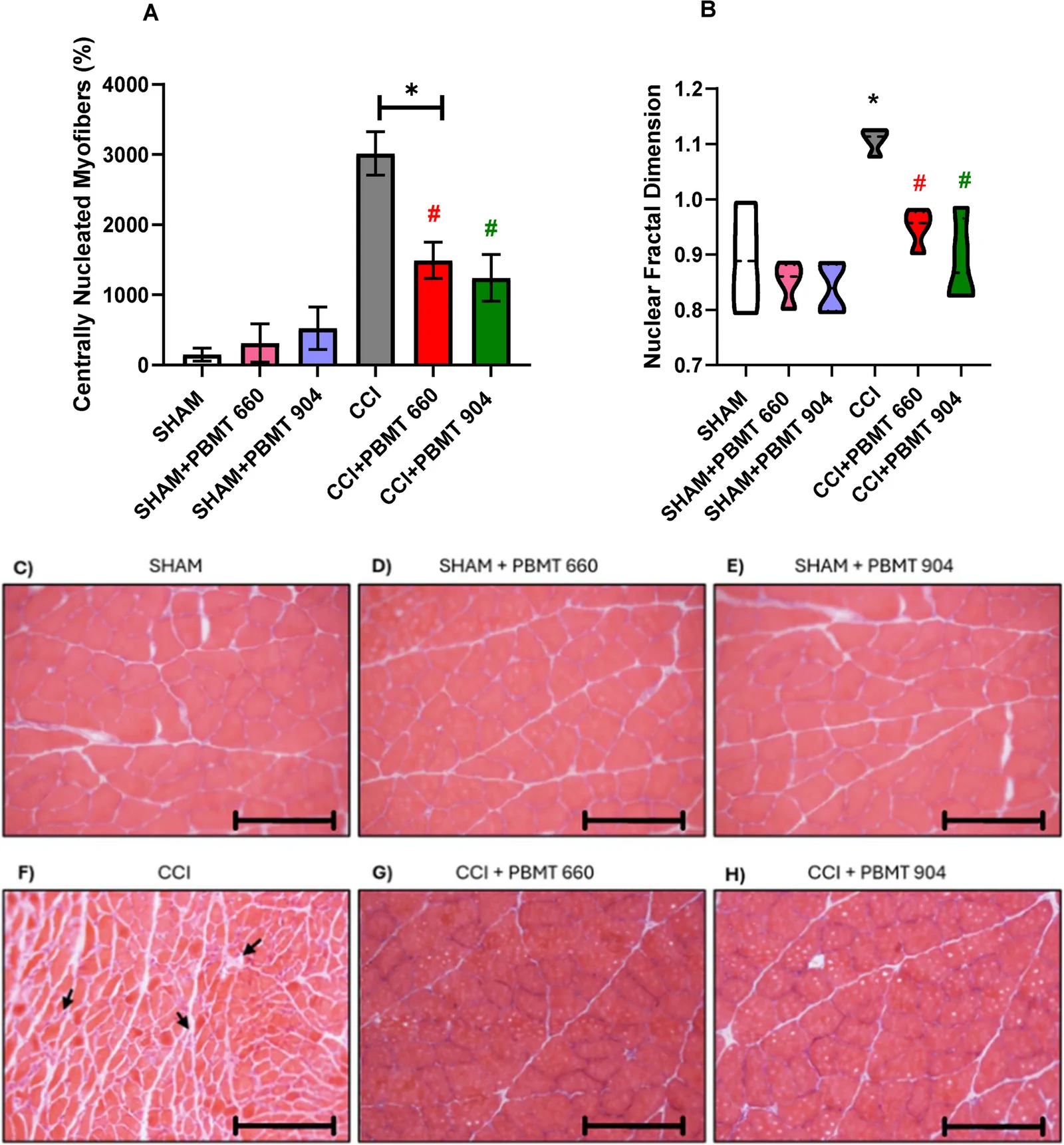
Photobiomodulation attenuates muscle fiber remodeling and structural disorganization induced by sciatic nerve constriction. (**A**) Quantification of centrally nucleated muscle fibers, expressed as percentage. PBMT significantly reduced the percentage of centrally nucleated fibers, with effects observed in both treated groups (CCI + PBMT 660: #*P* < 0.01; CCI + PBMT 904: #*P* < 0.001). (**B**) Nuclear fractal dimension analysis of gastrocnemius muscle fibers. The CCI group showed significantly higher fractal dimension values, indicating increased structural disorganization, compared with control groups (*P* < 0.001). Both PBMT protocols significantly reduced fiber disorganization relative to untreated CCI animals (CCI + PBMT 660: #*P* < 0.01; CCI + PBMT 904: #*P* < 0.001). (**C**) Representative cross-sections of gastrocnemius muscle stained with hematoxylin and eosin (H&E; 100× magnification. Data are expressed as mean ± S.E.M. and were analyzed by one-way ANOVA followed by post hoc testing. *n* = 7 animals per group. Scale bar = 100 μm

CCI animals exhibited a significant increase in the proportion of centrally nucleated muscle fibers compared with control groups (SHAM, SHAM + PBMT 660, and SHAM + PBMT 904; Fig. 5A; F=(5, 20) = 14,91, *P < 0.0001), indicating ongoing muscle remodeling associated with nerve injury. Treatment with both red (660 nm) and infrared (904 nm) photobiomodulation significantly reduced the percentage of centrally nucleated myofibers relative to the untreated CCI group (Fig. 5A), suggesting attenuation of injury-induced muscle alterations. Notably, infrared PBMT (CCI + PBMT 904; #*P* < 0.001) was more effective than red PBMT (CCI + PBMT 660; #*P* < 0.01) with a 59% reduction in inflammatory cells, while the red laser showed a 51% reduction, a finding consistent with the limited attenuation of muscle atrophy observed in the 660 nm group when compared to the CCI group (Fig. 5).

Nuclear fractal dimension analysis, which reflects the degree of cellular and tissue disorganization, with values closer to 2 indicating greater disorganization, revealed a marked increase in muscle fiber disorganization following sciatic nerve constriction (Fig. 5B). The CCI group displayed significantly higher fractal dimension values compared with control groups (F (5, 18) = 12,87, *P* < 0.0001). Importantly, both PBMT protocols significantly reduced fiber disorganization relative to untreated CCI animals (CCI + PBMT 660: #*P* < 0.001; CCI + PBMT 904: #*P* < 0.001), with a more pronounced effect observed in the infrared-treated group.

These quantitative findings are supported by representative histological images (Fig. 5C-H), in which CCI muscles exhibit inflammatory infiltrates, fiber atrophy, and marked structural disorganization as indicated by the arrows in the Fig. 5F. In contrast, muscles from PBMT-treated animals display improved fiber organization and morphology more closely resembling control groups. Taken together, these results indicate that photobiomodulation therapy mitigates injury-induced muscle remodeling and preserves gastrocnemius muscle structural integrity following chronic sciatic nerve constriction.

### Impact of photobiomodulation on mitochondrial function in skeletal muscle

Macro and microscopical characterizations showed how PBMT improves morphological characteristics in the muscle, so the next experiment follows the organellar (mitochondrial state) after the treatments by oxygen consumption analyses. Light can modulate mitochondrial respiration, for that reason we performed mitochondrial isolation from gastrocnemius muscle and oxygen consumption were assessed using high-resolution Oroboros O2k system. Sequential and calibrated compound addition allowed evaluation of respiratory states: rotenone and succinate for basal respiration, ADP (State 3), oligomycin for complex V inhibition (State 4), and CCCP for maximal uncoupled respiration (State 3U).

**Fig. 6:**
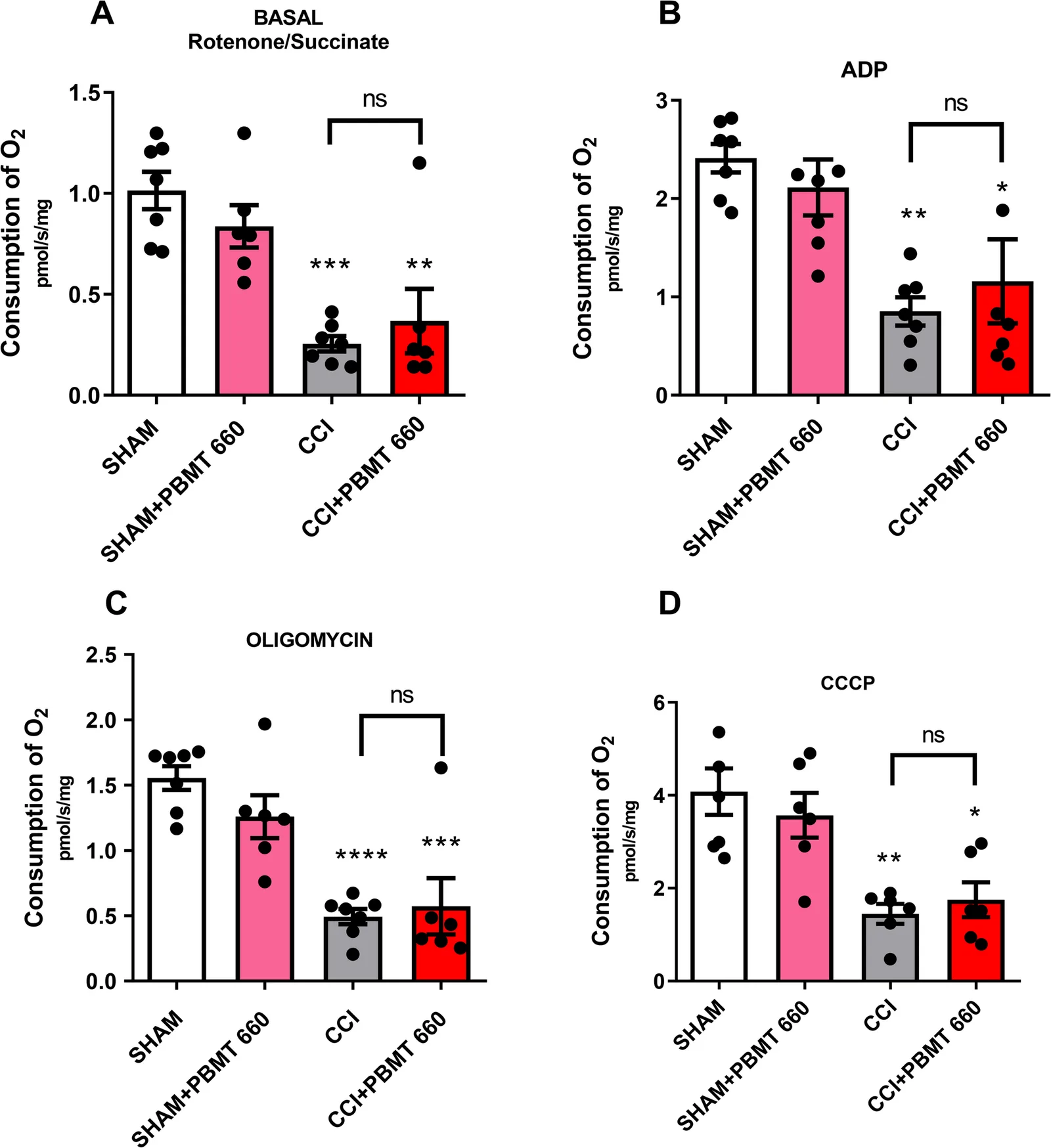
Mitochondrial respiration analyses in animals treated with PBMT 660 nm. High-resolution respirometry of gastrocnemius muscle mitochondria in SHAM, SHAM + PBMT 660, CCI, and CCI + PBMT 660 nm groups. All titrated modulators as 1 µM Rotenone + 1 mM succinate (A, state 2), 1 mM ADP (B, state 3), 1 µM oligomycin (C, Oligo, state 4), and 1 mM CCCP (D, state 3U) were added sequentially while oxygen consumption was quantified as described in materials and methods. CCI markedly reduced mitochondrial oxygen consumption compared with SHAM, while PBMT 660 nm showed no significant recovery, but partially narrowed the gap relative to SHAM. Data expressed as mean ± SEM. *P < 0.05; **P < 0.01; ***P < 0.001 and ****P < 0.0001 vs. SHAM and ns represents non-statistical significance. The number of replicates per group was *n* = 7

**Fig. 7:**
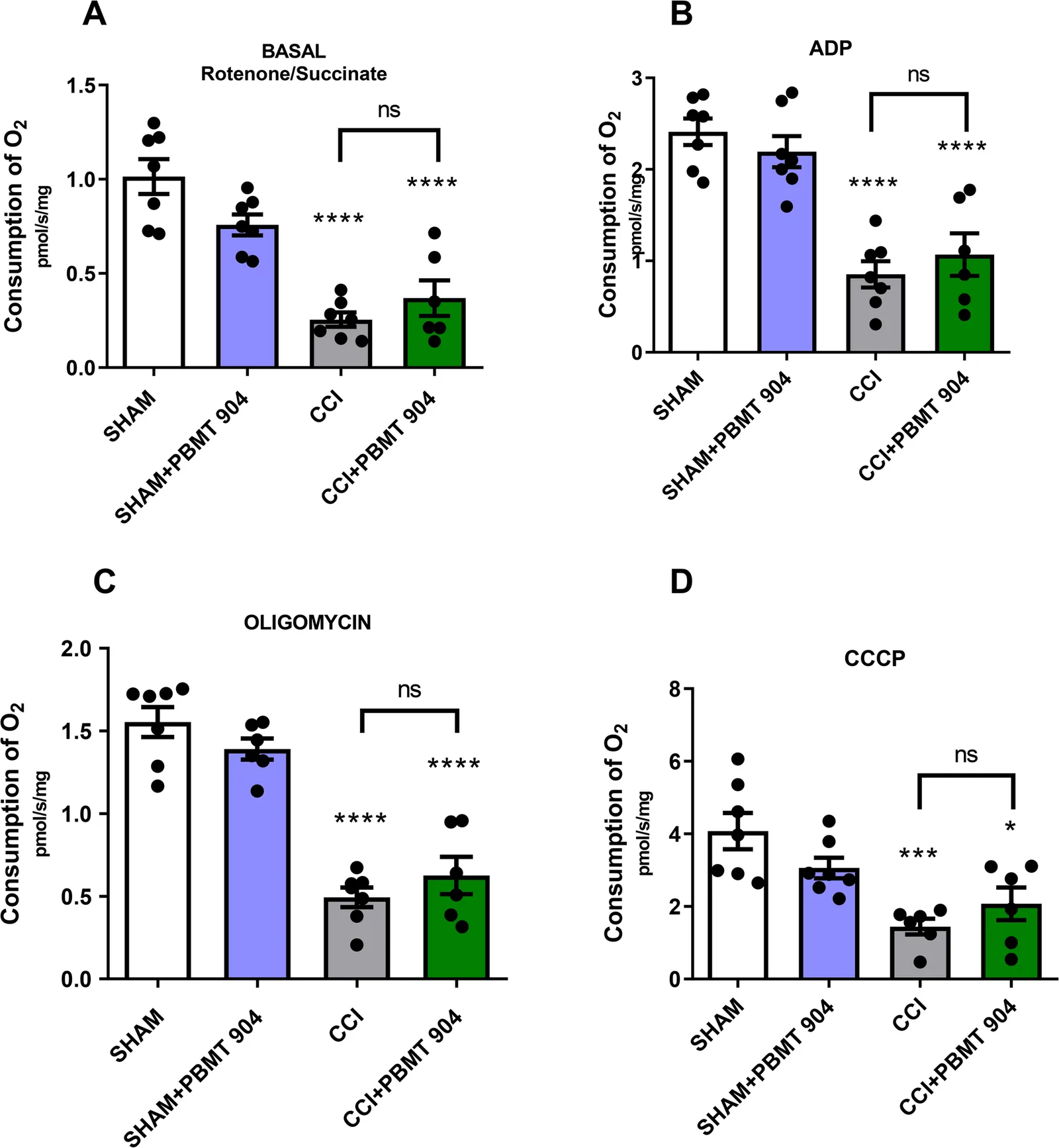
Mitochondrial respiration analyses in animals treated with PBMT 904 nm. High-resolution respirometry of gastrocnemius muscle mitochondria in SHAM, SHAM + PBMT 904, CCI, and CCI + PBMT 904 nm groups. All titrated modulators as 1 µM Rotenone + 1 mM succinate (A, state 2), 1 mM ADP (B, state 3), 1 µM oligomycin (C, Oligo, state 4), and 1 mM CCCP (D, state 3U) were added sequentially while oxygen consumption was quantified as described in materials and methods. In these results the same control as with PBMT 660 nm was used. CCI significantly decreased respiration compared with SHAM, and PBMT 904 nm did not fully restore values but showed a trend toward improvement, reducing the difference relative to SHAM. Data expressed as mean ± SEM. *P < 0.05; **P < 0.01; ***P < 0.001 and ****P < 0.0001 vs. SHAM and ns represent non-statistical significance

In animals subjected to sciatic nerve constriction (CCI), mitochondrial respiration was significantly reduced across all respiratory states compared with SHAM controls (Figs. 6 and 7 for Red and Infrared, respectively). For PBMT at 660 nm, no statistically significant recovery was detected when compared with untreated CCI, although a biological trend toward higher oxygen consumption was observed (Fig. 6A- F (3, 22) = 13,00; 6B- F (3, 24) = 7,287; 6 C- F (3, 22) = 14,84; 6D- F (3, 21) = 9,895). Importantly, the difference between CCI + PBMT 660 and SHAM (*P* < 0.001) was smaller than between CCI and SHAM (*P* < 0.0001), suggesting a partial modulatory effect.

Similar findings were obtained in the 904 nm group, where no significant differences were found between CCI and CCI + PBMT 904, but respiration rates tended to improve, again reducing the gap relative to SHAM animals (Fig. 7A- F (3, 23) = 23,71; 7B- F (3, 23) = 21,12; 7 C- F (3, 22) = 41,94; 7D- F (3, 22) = 8,924). Together, these results indicate that CCI impairs mitochondrial respiratory capacity in skeletal muscle, and although PBMT at both 660 nm and 904 nm did not fully restored respiration as expected in the hypothesis.

The percentage analysis of the results highlights the effect of photobiomodulation at both wavelengths is represented in Table 2. Overall, it can be observed that both wavelengths were able to attenuate the reductions promoted by the injury, with PBMT 904 nm being particularly more effective in graph D and PBMT 660 nm in graph B.

**Table 2:** Percentage of reduction in oxygen consumption rates compared to control group. Respiration in the control group (SHAM) was used as a relative reference to compare individual differences on mitochondrial states by percentages in the basal respiration, ADP, Oligomycin and CCCP presence

Group	Mitochondrial state (Graph)	Percentage of reduction compared to control group- SHAM (100%)
CCI	Basal (6 A e 7 A)	75%
CCI + PBMT 660	Basal (6 A)	64%
CCI + PBMT 904	Basal (7 A)	64%
CCI	ADP (6B e 7B)	65%
CCI + PBMT 660	ADP (6B)	52%
CCI + PBMT 904	ADP (7B)	56%
CCI	Oligomycin (6 C e 7 C)	69%
CCI + PBMT 660	Oligomycin (6 C)	64%
CCI + PBMT 904	Oligomycin (7 C)	60%
CCI	CCCP (6D e 7D)	65%
CCI + PBMT 660	CCCP (6D)	58%
CCI + PBMT 904	CCCP (7D)	50%

#### Evaluation of PBMT by mitochondrial protein expression in a model of sciatic nerve chronic constriction injury

**Fig. 8:**
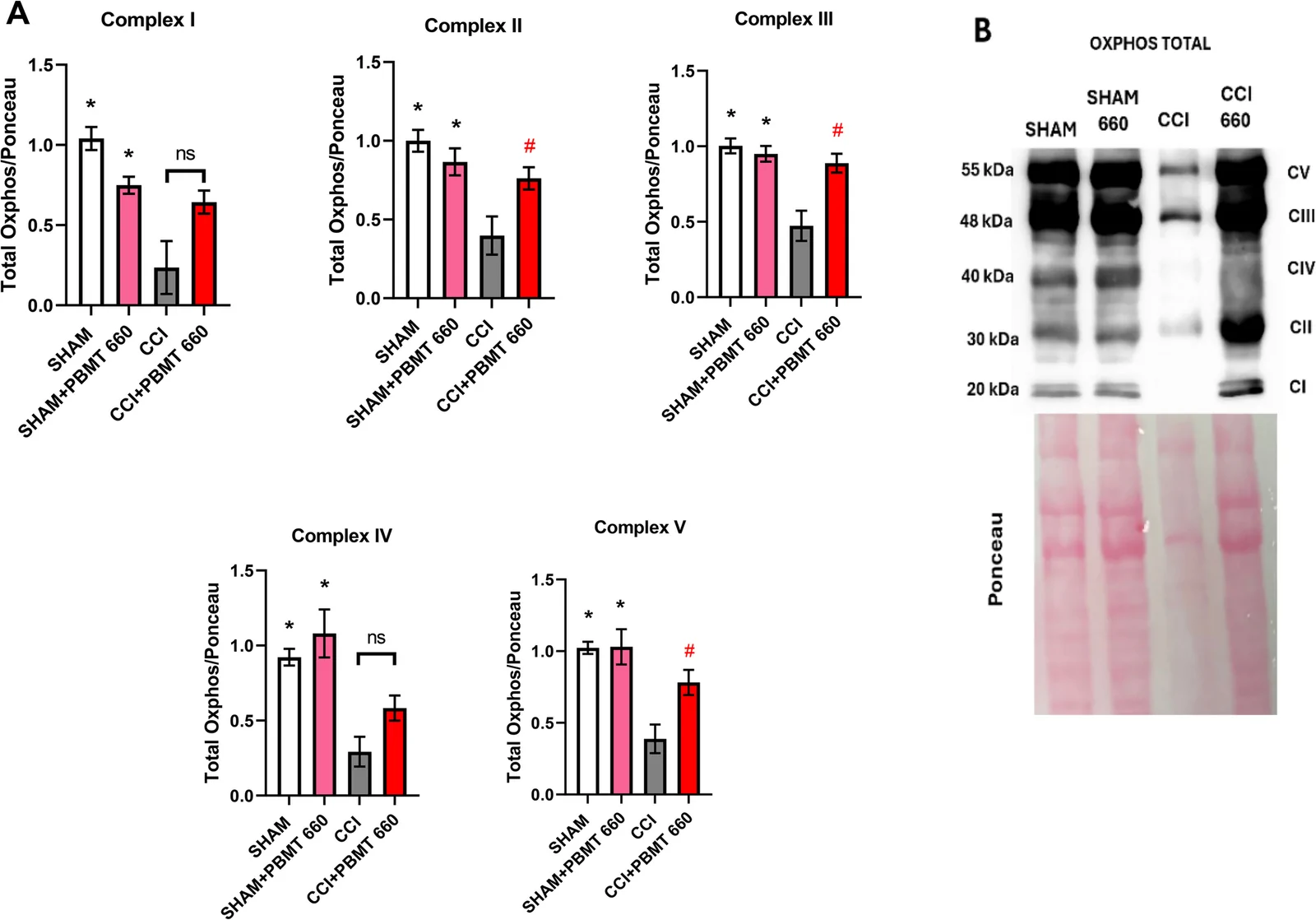
PBMT at 660 nm enhances protein expression of mitochondrial complexes II, III, and V in a CCI model. Panel A shows the experimental groups treated with red laser (660 nm). The symbol *P < 0.01 indicates differences between the CCI group and the control groups (SHAM and SHAM + PBMT 660 nm), observed across all graphs. No statistically significant differences were detected for complexes I and IV. Significant increases were observed for complex II (#*P* < 0.05), complex III (#*P* < 0.001), and complex V (#*P* < 0.05), representing differences between the CCI and CCI + PBMT 660 nm groups. Data are expressed as mean ± S.E.M. and analyzed by one-way ANOVA

**Fig. 9:**
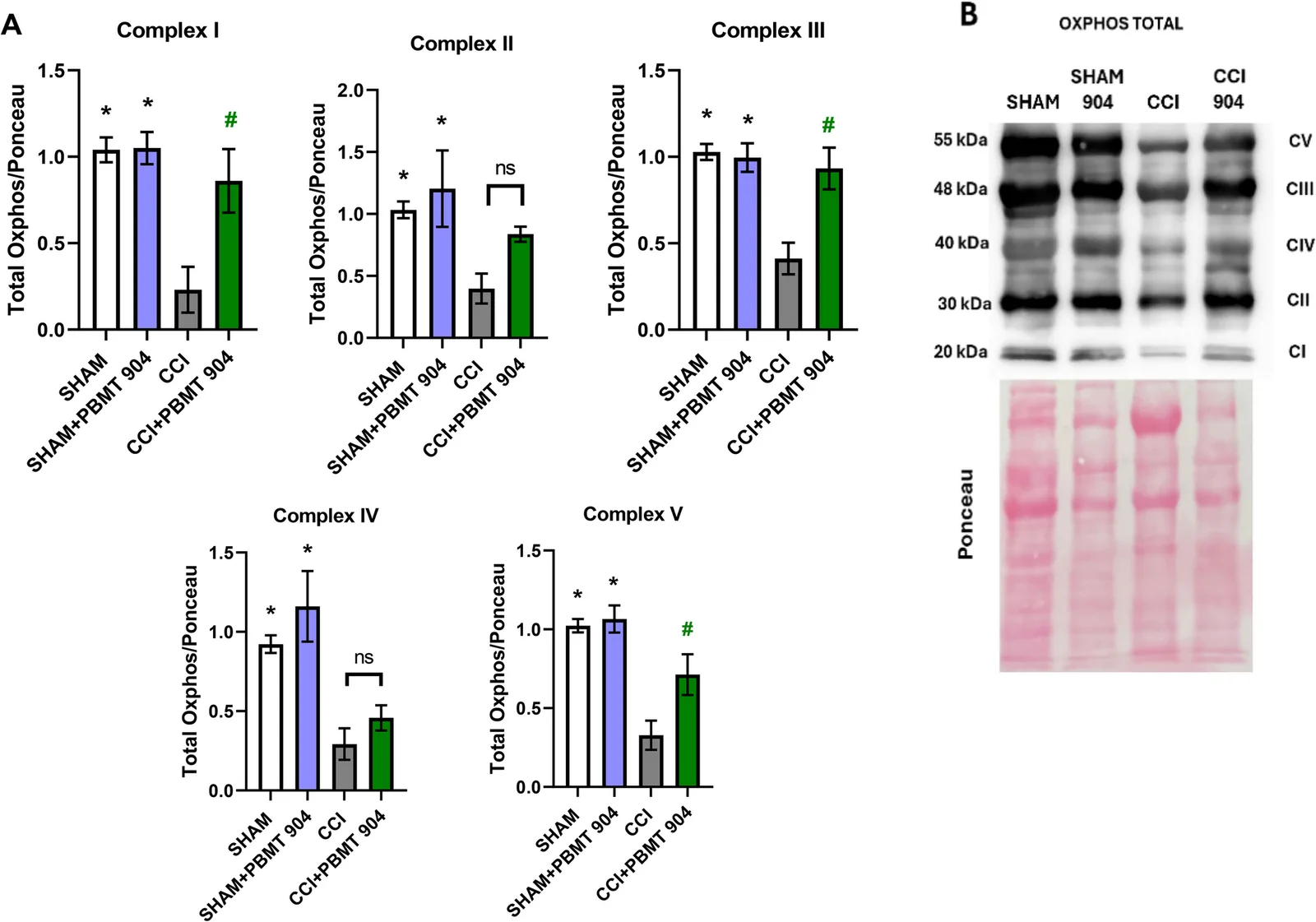
PBMT at 904 nm enhances protein expression of mitochondrial complexes I, III, and V in a CCI model. Panel A shows the experimental groups treated with infrared laser (904 nm). The symbol *P < 0.01 indicates differences between the CCI group and the control groups (SHAM and SHAM + PBMT 904 nm), observed across all graphs. No statistically significant differences were detected for complexes II and IV. Significant increases were observed for complex I (#*P* < 0.001), complex III (#*P* < 0.001), and complex V (#*P* < 0.05), representing differences between the CCI and CCI + PBMT 904 nm groups. Data are expressed as mean ± S.E.M. and analyzed by one-way ANOVA

**Fig. 10:**
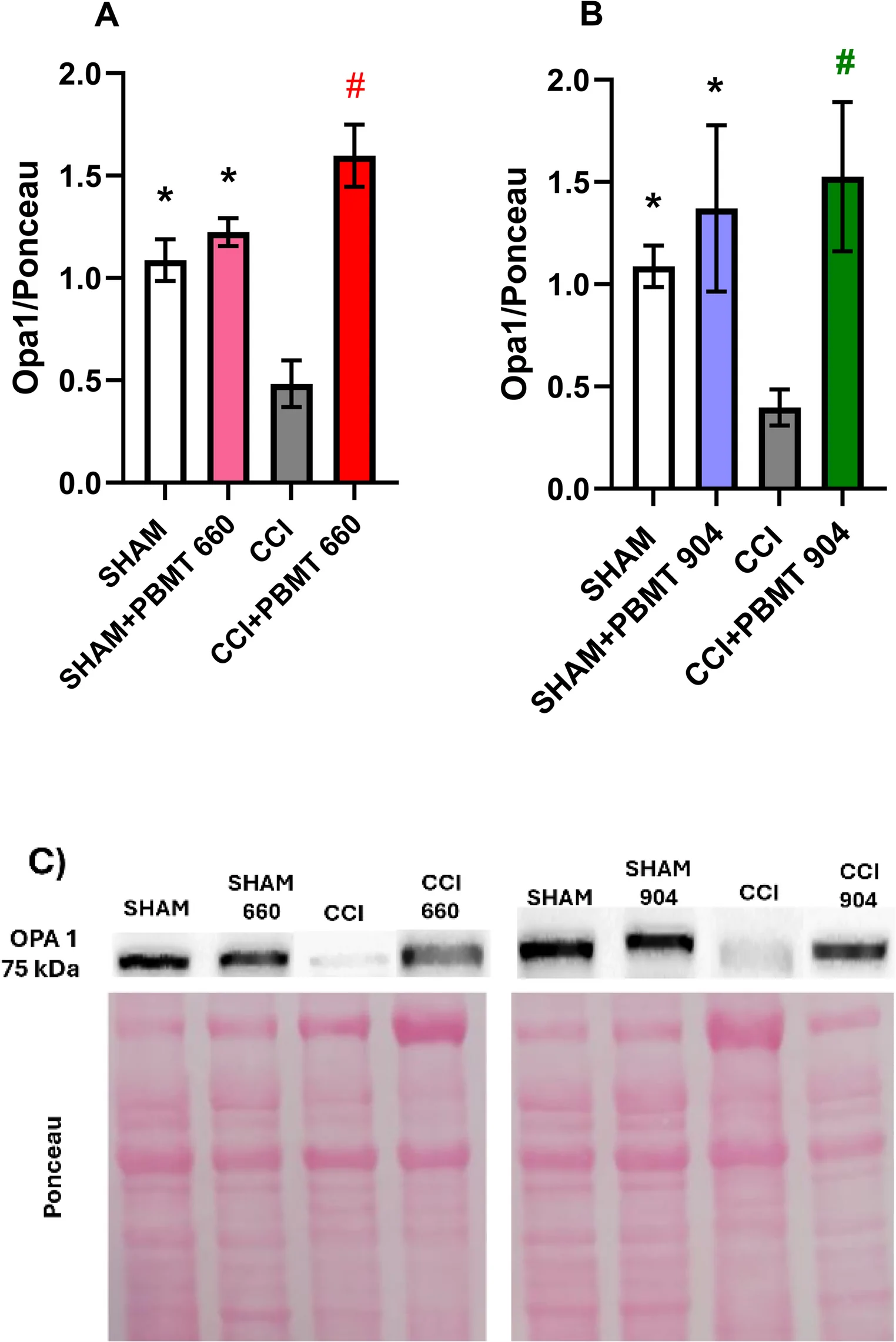
Analysis of protein expression in isolated mitochondria from gastrocnemius muscle, using the OPA1 antibody. Figure A represents the group treated with red laser (660 nm), and Figure B represents the group treated with infrared laser (904 nm), where *P < 0.01 indicates the difference between the control groups (SHAM, SHAM + PBMT 660, and SHAM + PBMT 904) and the CCI group. Figure A – #*P* < 0.0001 indicates the difference between the CCI and CCI + PBMT 660 groups. Figure B – #*P* < 0.05 indicates the difference between the CCI and CCI + PBMT 904 groups. Data are expressed as mean ± S.E.M. and were analyzed by one-way ANOVA

To investigate why PBMT failed to improve oxygen consumption in isolated skeletal muscle mitochondria following chronic constriction injury (CCI) of the sciatic nerve, we first examined the protein expression of mitochondrial respiratory chain complexes (OXPHOS subunits) and OPA by Western blotting (Figs. 8, 9 and 10).

In CCI animals treated with PBMT, in both wavelengths, we observed a significant increase in the expression of complex III (Q–cytochrome c oxidoreductase; UQCRC) and complex V (ATP synthase; ATP5A), whereas no changes were detected in complex IV (cytochrome c oxidase subunit II; COX II) (Figs. 8 and 9, respectively). Notably, the effects of PBMT on mitochondrial protein expression differed according to the wavelength applied. Treatment with red light (660 nm) selectively increased the expression of complex II (succinate dehydrogenase; SDHB) (Fig. 8A), whereas infrared PBMT (904 nm) selectively enhanced complex I expression (Fig. 9A). In contrast, complexes III and V were consistently upregulated by both wavelengths.

Mitochondrial respiration can be modulated by the mitochondrial morphology, for that reason we performed a screening of OPA1. Significant reduction in OPA1 expression was observed in the CCI group compared to all control groups (SHAM, SHAM + PBMT 660, and SHAM + PBMT 904; *P < 0.01). Figure 10A and B, corresponding to red (660 nm) and infrared (904 nm) PBMT treatments, respectively, illustrate OPA1 protein expression levels. Moreover, when compared to the PBMT-treated CCI groups, the untreated CCI group exhibited significantly lower OPA1 protein expression (CCI + PBMT 660, #*P* < 0.0001; CCI + PBMT 904, #*P* < 0.05), indicating that PBMT partially restored OPA1 levels in neuropathic animals.

## Discussion

Neuropathic pain represents a major public health challenge due to its high prevalence, negative impact on quality of life, and the limited therapeutic efficacy of currently available pharmacological treatments, which are frequently associated with adverse effects and an increased risk of psychiatric complications [4, 5]. These limitations reinforce the need for non-pharmacological approaches, such as photobiomodulation therapy (PBMT), which has been extensively investigated for its analgesic effects and its ability to modulate cellular processes involved in pain [50]. In this context, the present study investigated the effects of red and infrared PBMT on neuropathic pain and the muscle and mitochondrial alterations induced by chronic constriction injury (CCI) of the sciatic nerve.

Behavioral analyses demonstrated that PBMT effectively increased nociceptive thresholds in mechanical hyperalgesia and tactile allodynia. Treatment with 904 nm PBMT produced an earlier antinociceptive effect, whereas the 660 nm wavelength induced significant improvements only during the later stages of the experimental protocol. These findings are consistent with previous studies demonstrating robust analgesic effects of infrared PBMT in neuropathic pain models and suggest that wavelength influences the temporal profile of analgesia [17, 51–56]. However, although infrared PBMT produced an earlier response, statistically significant differences between the two wavelengths were not consistently observed across all evaluated outcomes. Therefore, the results suggest a more consistent performance of infrared PBMT for certain parameters but do not support the conclusion that this wavelength is universally superior to red PBMT. In contrast, thermal hyperalgesia was not significantly attenuated by either wavelength. This lack of effect may be related to the delayed initiation of treatment (beginning on day 14 after injury), as earlier interventions and different dosing regimens have been shown to be more effective in modulating thermal sensitivity [24, 57–59]. Infrared thermography revealed a reduction in local temperature within the irradiated areas, indicating that PBMT-induced analgesia was not mediated by thermal effects. These findings support the concept that PBMT acts through photochemical and photobiological mechanisms rather than tissue heating, in agreement with recent literature emphasizing the importance of distinguishing photobiomodulation from photothermal effects [60].

Beyond nociceptive modulation, CCI induced marked alterations in the gastrocnemius muscle, including muscle mass reduction, inflammatory cell infiltration, and muscle fiber disorganization [25, 61, 62]. The results demonstrated that 904 nm PBMT partially attenuated muscle mass loss, whereas treatment with 660 nm PBMT did not significantly affect this parameter under the experimental conditions evaluated. On the other hand, both wavelengths reduced inflammatory cell infiltration and improved muscle fiber organization. Considering the close relationship between peripheral inflammation, tissue damage, and nociceptive sensitization, these alterations may have contributed to the behavioral improvements observed. However, it is important to emphasize that the present study evaluated only histological alterations and did not investigate inflammatory cytokines or molecular signaling pathways involved in the inflammatory process.

Another aspect that deserves consideration is that the partial preservation of muscle morphology observed following PBMT may reflect not only a direct effect of the therapy on skeletal muscle tissue but also an indirect effect resulting from pain reduction. Improvement in nociceptive behavior may promote greater functional use of the affected limb, thereby reducing muscle disuse and consequently contributing to the structural preservation observed. Therefore, although the present findings indicate a protective effect of PBMT on skeletal muscle, it is not possible to completely distinguish the direct effects of the therapy from those secondary to the improved functional use of the affected limb.

Furthermore, to the best of our knowledge, this is one of the first studies to simultaneously demonstrate the effects of PBMT on muscle structural organization, inflammatory cell infiltration, and mitochondrial function in skeletal muscle following chronic constriction injury of the sciatic nerve, thereby contributing to a broader understanding of the peripheral consequences of neuropathic pain beyond the nervous system.

Mitochondrial dysfunction has increasingly been recognized as a key contributor to neuropathic pain and peripheral tissue impairment [63–66]. In the present study, mitochondria isolated from the gastrocnemius muscle of CCI animals exhibited reduced respiratory activity, indicating that sciatic nerve injury compromises muscle bioenergetics. Considering the high energy demand of skeletal muscle, this impairment may contribute to the structural and functional alterations observed following partial denervation. Muscle atrophy itself may further exacerbate mitochondrial dysfunction, creating a vicious cycle of energetic impairment and functional decline. Although the use of isolated mitochondria represents a reductionist approach that may limit interactions with other cellular components, this method allowed direct assessment of mitochondrial respiratory capacity in the affected muscle tissue [67].

Experimental evidence indicates that photobiomodulation can modulate mitochondrial oxidative metabolism and the activity of respiratory chain complexes in different cell types, including muscle and neural cells, thereby influencing ATP production and redox signaling pathways associated with pain modulation [68–70]. In this context, we analyzed the protein expression of mitochondrial respiratory chain complexes to better understand the effects of PBMT on muscle bioenergetics following sciatic nerve constriction.

Our findings demonstrate that chronic constriction of the sciatic nerve significantly reduced the protein expression of mitochondrial complexes I–V, reinforcing the presence of marked mitochondrial impairment in this experimental model. PBMT partially reversed these alterations in a wavelength-dependent manner. Red PBMT increased the expression of complexes II, III, and V, whereas infrared PBMT enhanced the expression of complexes I, III, and V. These complexes are directly involved in oxidative metabolism, electron transfer, and ATP synthesis [71–73], suggesting that PBMT was able to induce relevant molecular adaptations in the mitochondrial machinery.

However, one of the most relevant findings of the present study was the dissociation between these molecular adaptations and mitochondrial function. Despite the increased expression of several respiratory chain complexes, neither treatment significantly restored the respiratory capacity of isolated mitochondria. These results indicate that increased protein abundance alone does not necessarily translate into functional recovery of oxidative phosphorylation. Mitochondrial activity depends on several factors beyond the expression of respiratory chain complexes, including the proper assembly of respiratory complexes and supercomplexes, their enzymatic activity, post-translational modifications, the integrity of the inner mitochondrial membrane, the cellular redox state, and the availability of metabolic cofactors [74–76]. Therefore, it is possible that PBMT induced early molecular adaptations that were not sufficient to fully restore mitochondrial bioenergetics under the experimental conditions evaluated.

Another factor that may have contributed to this dissociation was the persistent reduction in complex IV expression observed in both PBMT-treated groups. Complex IV represents the terminal step of the electron transport chain, being responsible for the final transfer of electrons to oxygen and playing a fundamental role in maintaining mitochondrial respiratory flux. Therefore, even in the presence of increased expression of complexes located upstream in the respiratory chain, the persistent impairment of complex IV may represent an important functional bottleneck, limiting the recovery of oxidative phosphorylation and ATP production [77].

In addition to the respiratory chain, we evaluated the expression of OPA1, an important regulator of inner mitochondrial membrane fusion and the maintenance of mitochondrial cristae architecture. PBMT increased OPA1 expression in animals with neuropathic pain, a finding consistent with studies reporting reduced OPA1 expression under conditions associated with mitochondrial fragmentation, impaired energy metabolism, and increased pain sensitivity [78]. However, although this increase suggests a possible modulation of processes related to mitochondrial fusion, the evaluation of OPA1 alone does not allow the conclusion that mitochondrial dynamics were restored. Other regulators of mitochondrial dynamics were not evaluated, precluding a comprehensive characterization of the mechanisms governing mitochondrial fusion and fission. Therefore, the present findings indicate that PBMT may influence specific components of mitochondrial dynamics but do not support the conclusion that this process was fully restored.

Taken together, these findings demonstrate that PBMT exerts beneficial effects on neuropathic pain-related behavior, muscle inflammation, structural organization, and mitochondrial protein expression, with infrared PBMT showing greater efficacy in nociceptive modulation and muscle preservation. However, chronic constriction injury of the sciatic nerve induces profound mitochondrial dysfunction that is only partially reversed by PBMT under the experimental conditions employed in the present study. By integrating behavioral, muscular, and mitochondrial analyses, this study advances the understanding of wavelength-dependent mechanisms of PBMT and highlights mitochondria as a critical, yet complex, therapeutic target in neuropathic pain.

In this context, future studies should investigate treatment protocols initiated at earlier stages after injury, different irradiation parameters, longer follow-up periods, and functional analyses of both neural and muscular tissues to better elucidate how the molecular alterations observed relate to the recovery of mitochondrial function and neuropathic pain.

## Conclusion

This study demonstrates that photobiomodulation therapy exerts wavelength-dependent effects on neuropathic pain and peripheral tissue damage. Infrared PBMT (904 nm) produced more sustained antinociceptive effects on mechanical hyperalgesia and tactile allodynia and partially preserved gastrocnemius muscle mass. In contrast, red PBMT (660 nm) produced later analgesic effects and did not significantly reduce muscle atrophy. Both wavelengths attenuated inflammatory cell infiltration and improved muscle fiber organization, indicating anti-inflammatory and tissue-protective effects. At the mitochondrial level, although neither wavelength reversed the marked overall impairment in respiratory capacity, both promoted molecular adaptations, including the upregulation of OPA1, a key regulator of mitochondrial dynamics, and the selective modulation of electron transport chain complexes. These findings suggest that PBMT can modulate specific components of the mitochondrial machinery; however, these alterations were not sufficient to restore mitochondrial oxygen consumption under the experimental conditions evaluated. Overall, this study makes an important contribution to scientific literature by advancing the understanding of the mechanisms underlying photobiomodulation, particularly by demonstrating a dissociation between functional mitochondrial respiration and beneficial molecular adaptations in skeletal muscle following peripheral nerve injury. The observed dissociation between molecular adaptations and mitochondrial function highlights the need for further studies to clarify how these alterations contribute to the analgesic effects and tissue recovery induced by PBMT, thereby supporting the optimization of treatment protocols and the development of future translational applications.

## Limitation of the study

Although the present findings demonstrate beneficial effects of PBMT on nociceptive behavior, muscle organization, and mitochondrial molecular adaptations, several limitations should be considered when interpreting the results. First, only male animals were included in the study, preventing extrapolation of the findings to females, given the well-recognized sex differences in the pathophysiology of neuropathic pain and in the response to therapeutic interventions. Furthermore, the analyses were restricted to the gastrocnemius muscle and did not include tissues more directly involved in the pathophysiology of neuropathic pain, such as the sciatic nerve or dorsal root ganglia. Future studies should investigate these tissues to provide a more comprehensive understanding of the peripheral and neural mechanisms underlying the effects of PBMT. Another limitation is that the evaluations were performed at a single experimental time point, at the end of the treatment protocol. Although this approach allowed the investigation of the late effects of PBMT, analyses at different time points following injury could provide a better understanding of the temporal progression of muscle, mitochondrial, and behavioral alterations, as well as identify when the molecular adaptations observed in the present study occur.

Despite these limitations, the present study provides an integrated approach by simultaneously investigating behavioral, structural, and mitochondrial alterations in skeletal muscle following peripheral nerve injury. The findings demonstrate that PBMT promotes important benefits in neuropathic pain and muscle remodeling while inducing molecular adaptations in mitochondria, even in the absence of complete recovery of mitochondrial respiratory capacity. These results reinforce the complexity of the mechanisms underlying the response to PBMT and suggest that molecular adaptations may precede the functional recovery of mitochondrial bioenergetics.

## Data Availability

The datasets generated and/or analyzed during the current study are available from the corresponding author on reasonable request.
